# A minimalist mitochondrial threonyl-tRNA synthetase exhibits tRNA-isoacceptor specificity during proofreading

**DOI:** 10.1093/nar/gku1218

**Published:** 2014-11-20

**Authors:** Xiao-Long Zhou, Zhi-Rong Ruan, Meng Wang, Zhi-Peng Fang, Yong Wang, Yun Chen, Ru-Juan Liu, Gilbert Eriani, En-Duo Wang

**Affiliations:** 1 State Key Laboratory of Molecular Biology, Institute of Biochemistry and Cell Biology, Shanghai Institutes for Biological Sciences, The Chinese Academy of Sciences, 320 Yue Yang Road, Shanghai, China; 2 School of Life Science and Technology, ShanghaiTech University, 319 Yue Yang Road, 200031 Shanghai, China; 3 Architecture et Réactivité de l'ARN, Université de Strasbourg, UPR9002 CNRS, Institut de Biologie Moléculaire et Cellulaire, 15 rue René Descartes, 67084 Strasbourg, France

## Abstract

Yeast mitochondria contain a minimalist threonyl-tRNA synthetase (ThrRS) composed only of the catalytic core and tRNA binding domain but lacking the entire editing domain. Besides the usual tRNA^Thr^2, some budding yeasts, such as *Saccharomyces cerevisiae*, also contain a non-canonical tRNA^Thr^1 with an enlarged 8-nucleotide anticodon loop, reprograming the usual leucine CUN codons to threonine. This raises interesting questions about the aminoacylation fidelity of such ThrRSs and the possible contribution of the two tRNA^Thr^s during editing. Here, we found that, despite the absence of the editing domain, *S. cerevisiae* mitochondrial ThrRS (*Sc*mtThrRS) harbors a tRNA-dependent pre-transfer editing activity. Remarkably, only the usual tRNA^Thr^2 stimulated pre-transfer editing, thus, establishing the first example of a synthetase exhibiting tRNA-isoacceptor specificity during pre-transfer editing. We also showed that the failure of tRNA^Thr^1 to stimulate tRNA-dependent pre-transfer editing was due to the lack of an editing domain. Using assays of the complementation of a *Sc*mtThrRS gene knockout strain, we showed that the catalytic core and tRNA binding domain of *Sc*mtThrRS co-evolved to recognize the unusual tRNA^Thr^1. In combination, the results provide insights into the tRNA-dependent editing process and suggest that tRNA-dependent pre-transfer editing takes place in the aminoacylation catalytic core.

## INTRODUCTION

Accurate transfer of genetic information is of critical significance for cellular function and maintenance. Several steps, including DNA replication, mRNA transcription and protein synthesis, contribute to high accuracy with different levels of fidelity (1). Protein synthesis is initiated by an ancient group of enzymes, the aminoacyl-tRNA synthetases (aaRSs) containing 20 members in the majority of living species (2–4). These enzymes catalyze the ligation of a specific amino acid to their specific tRNA-isoacceptors. This reaction, aminoacylation, is performed by most aaRSs in two successive steps. The first step involves adenosine triphosphate (ATP)-dependent amino acid activation in which an intermediate aminoacyl-(adenosine monophosphate (AMP)) is generated with the release of pyrophosphate. This is followed by the transfer of the activated amino acid moiety from the aminoacyl-AMP to the tRNA (2).

Protein synthesis, which is the last step in the expression of the genetic code, has a very high level of global fidelity, with a mis-incorporation of only one in every 10 000 codons under normal growth conditions (1). This high level of fidelity is challenging for some aaRSs, which have to discriminate between different amino acids and metabolites that can be structurally and chemically very similar. This critical paradox has been solved by the evolution of the proofreading (editing) function of some error-prone tRNA synthetases (5,6). Editing is critical for translational quality control and its impairment or abolition leads to ambiguities in the genetic code and serious cellular dysfunction (7,8). The ‘double sieve mechanism’ has been proposed to control editing function, in which only mis-activated non-cognate amino acids are removed, while access of the cognate residue to the editing active site is blocked by steric exclusion (9). Editing activity is based on the hydrolysis of mis-activated aminoacyl-AMPs (pre-transfer editing) and/or the hydrolysis of mis-charged aminoacyl-tRNAs (post-transfer editing) (5). Pre-transfer editing can be further divided into tRNA-independent and tRNA-dependent types according to whether the aminoacyl-AMP hydrolysis is stimulated by tRNA. Post-transfer editing usually takes place in a separated editing domain, such as the CP1 domain in class Ia aaRSs and the N2 domain of class II threonyl-tRNA synthetase (ThrRS) (5,6). Furthermore, tRNA-independent pre-transfer editing is believed to occur in the aminoacylation domain, as illustrated by the hydrolysis of aminoacyl-AMP by glutaminyl-tRNA synthetase (GlnRS), seryl-tRNA synthetase (SerRS), prolyl-tRNA synthetase (ProRS) and CP1-deprived leucyl-tRNA synthetase (LeuRS) (for a review see (5,6)). In contrast, the location of the tRNA-dependent pre-transfer editing site is still under debate and several controversial reports suggest that it is located in the editing domain (10–16) or in the aminoacylation domain (17–19). In addition, most natural aaRSs exhibiting tRNA-dependent pre-transfer editing capacity also possess an editing domain to catalyze post-transfer editing, thus, further complicating the assignment of the active site of tRNA-dependent pre-transfer editing.

The mitochondrion has its own translational system, producing several protein components of respiratory complexes. AaRSs for mitochondrial translation are usually encoded by the nuclear genome and then transported into the mitochondrion; however, most tRNAs are encoded by the mitochondrial genome (20). For instance, human mitochondria express 22 tRNA-isoacceptors corresponding to 20 amino acids with two tRNAs decoding serine [tRNA^Ser^(AGY) and tRNA^Ser^(UCN)] or leucine [tRNA^Leu^(CUN) and tRNA^Leu^(UUR)] (http://mamit-trna.u-strasbg.fr/Summary.asp). The mitochondrial genome of *Saccharomyces cerevisiae* encodes 24 tRNA-isoacceptors that decode all codons, including two tRNAs^Arg^, tRNAs^Ser^, tRNAs^Thr^ and tRNAs^Met^. However, *S. cerevisiae* mitochondria express only tRNA^Leu^(UUR) without tRNA^Leu^(CUN) (21). The mechanism underlying the translational quality control of the mitochondrial system is an interesting issue since it directly regulates the precise flow of the mitochondrial genetic code. Human mitochondrial leucyl-tRNA synthetase (hmtLeuRS) has been reported to be defective in post-transfer editing because of a degenerate CP1 domain; however, it has a more rigorous amino acid activation site to exclude non-cognate amino acids (22). Similarly, in contrast to its bacterial and eukaryotic cytoplasmic counterparts, yeast mitochondrial phenylalanyl-tRNA synthetase (PheRS) harbors no editing domain but selects Phe over Tyr more efficiently (23).

**Figure 1.**
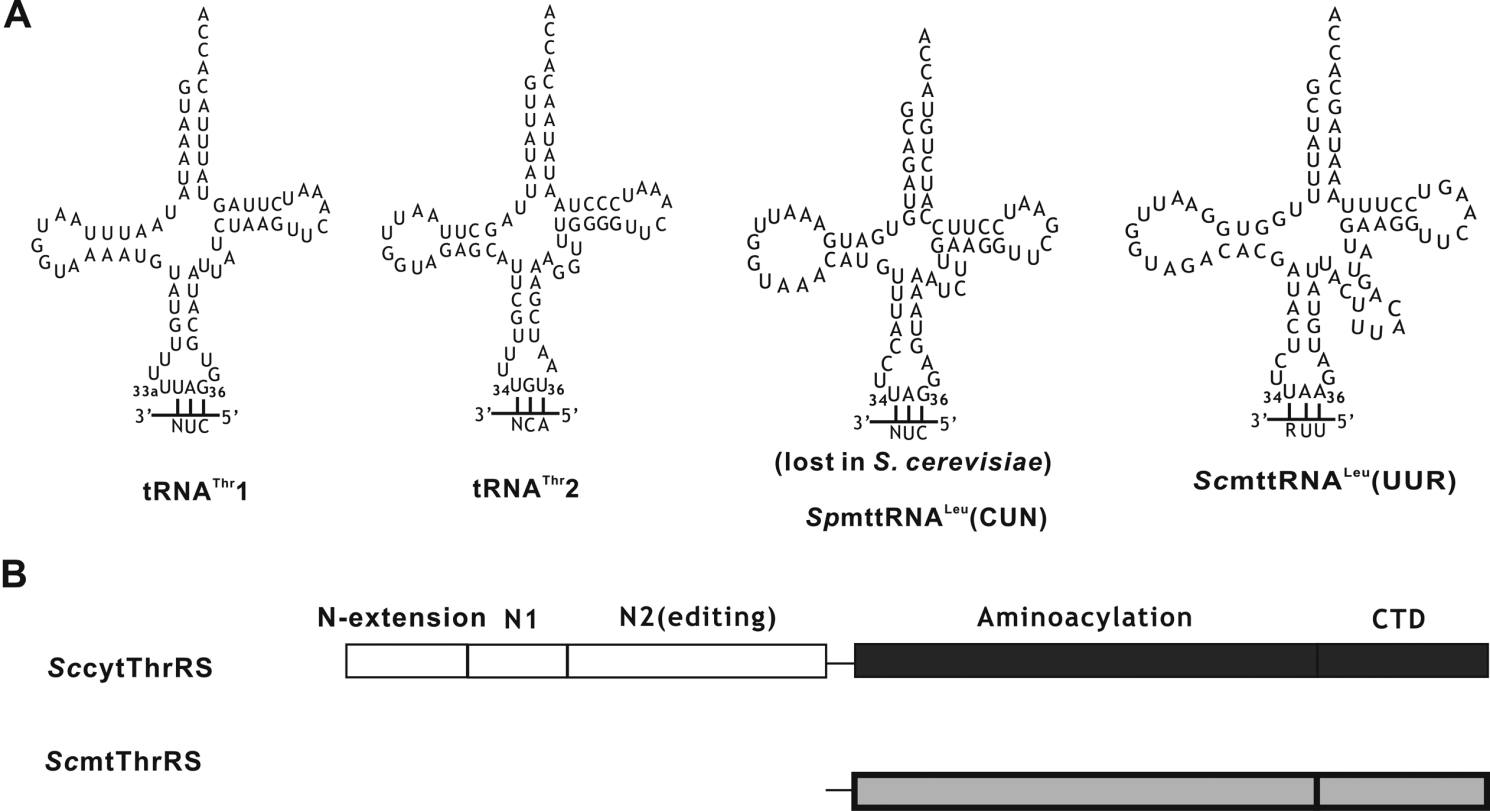
Representations of tRNAs and *S. cerevisiae* ThrRSs investigated in this study. (A) Cloverleaf structures of *S. cerevisiae* mitochondrial tRNA^Thr^1, tRNA^Thr^2, tRNA^Leu^(UUR) [*Sc*mttRNA^Leu^(UUR)] and *S. pombe* mitochondrial tRNA^Leu^(CUN) [*Sp*mttRNA^Leu^(CUN)], which has been lost in *S. cerevisiae* mitochondria during evolution. (B) Linear representation of the domain arrangement of *Sc*cyt*ThrRS* and *Sc*mtThrRS. Aminoacylation domain and CTD of *Sc*cyt*ThrRS* or *Sc*mtThrRS are colored in black or gray, respectively.

Components of the translational machinery of some budding yeasts, such as *S. cerevisiae*, display unique characteristics. First, the canonical leucine (Leu) codon CUN (N: A, G, C, T) is reassigned to threonine (Thr) in the *S. cerevisiae* mitochondrion (24), although the evolutionary benefit of this reassignment is unclear. This reassignment is mediated by a structurally unique *S. cerevisiae* mitochondrial tRNA^Thr^1 (tRNA^Thr^1) with an enlarged anticodon loop containing the ^34^UAG^36^ anticodon. The loop enlargement is due to the insertion of U between U33 and the ^34^UAG^36^ anticodon, which is designated as U33a here (Figure 1A) (25). The ^34^UAG^36^ anticodon is harbored by mitochondrial tRNA^Leu^(CUN) in other organisms, such as humans and even the yeasts *Schizosaccharomyces pombe*, *Candida albicans*. tRNA^Leu^(CUN) has been consistently lost in the *S. cerevisiae* mitochondrion during the evolution of the 24 mitochondrial tRNA genes (Figure 1A) (26). However, phylogenetic and biochemical data show that, in fact, tRNA^Thr^1 is not derived from the lost tRNA^Leu^ (CUN) but from tRNA^His^ with a ^34^GUG^36^ anticodon (27). In addition, a canonical *S. cerevisiae* mitochondrial tRNA^Thr^2 (tRNA^Thr^2) with the ^34^UGU^36^ anticodon decodes normal ACN Thr codons in the *S. cerevisiae* mitochondrion (Figure 1A) (25). Second, the enzyme catalyzing the aminoacylation of tRNA^Thr^1 and tRNA^Thr^2, *S. cerevisiae* mitochondrial ThrRS (*Sc*mtThrRS), encoded by the *MST1* gene, is devoid of an editing domain, and consists only of the aminoacylation catalytic core connected to the C-terminal tRNA binding domain (CTD) (Figure 1B) (28,29). This phenomenon also occurs in the mitochondria of other yeasts, such as *S. pombe* and *C. albicans*, although the CUN codons still encode Leu. This phenomenon suggests that loss of the editing domain occurred at a very early stage in the evolution of yeast, while CUN reassignment was a more recent event. In contrast, bacterial, eukaryotic cytoplasmic and other mitochondrial ThrRSs contain a N2 editing domain that hydrolyzes mis-charged Ser-tRNA^Thr^ (19,30). *Sc*mtThrRS has been reported to mis-activate Ser and to use tRNA-independent pre-transfer editing to remove Ser-AMP (31). In addition, only *Sc*mtThrRS, but not *S. pombe* or *C. albicans* mitochondrial ThrRS (*Sp*mtThrRS or *Ca*mtThrRS), recognizes tRNA^Thr^1, suggesting the evolution of tRNA^Thr^1 recognition elements in *Sc*mtThrRS, which have yet to be identified (27). Furthermore, whether other ThrRSs, such as bacterial and eukaryotic cytoplasmic, and mitochondrial ThrRSs can recognize the unique tRNA^Thr^1 is also unclear. Above all, investigation of the aminoacylation and editing mediated by *Sc*mtThrRS/tRNA^Thr^ is an interesting model with the two partners developing significant peculiarities during evolution.

In the present study, we showed that *Sc*mtThrRS exhibits a tRNA-dependent pre-transfer editing activity that is specific for the tRNA ^Thr^2 isoacceptor, whereas tRNA^Thr^1 was unable to stimulate such activity. We further confirmed the editing capability of tRNA^Thr^1, but demonstrated a requirement for the presence of an editing domain. We also identified the editing determinants of tRNA^Thr^2 and the editing antideterminants of tRNA^Thr^1. Finally, we constructed a yeast *MST1* gene knockout strain and, using a plasmid shuffle assay and different chimeric constructs, we showed that the catalytic core and tRNA binding domain of *Sc*mtThrRS co-evolved to recognize the unusual tRNA^Thr^1. In combination, the results of the present study provide insights into the tRNA-dependent editing process and also suggest that tRNA-dependent pre-transfer editing takes place in the aminoacylation catalytic core.

## MATERIALS AND METHODS

### Materials

L-Thr, L-Ser, dithiothreitol, ATP, CTP, GTP, UTP, 5′-GMP, tetrasodium pyrophosphate, inorganic pyrophosphate, Tris-HCl, MgCl_2_, NaCl and activated charcoal were purchased from Sigma (St. Louis, MO, USA). [^14^C]Thr was obtained from Biotrend Chemicals (Destin, FL, USA); [^14^C]Ser and [α-^32^P]ATP were obtained from Perkin Elmer Inc. (Waltham, MA, USA). The DNA fragment rapid purification kits and plasmid extraction kits were purchased from YPH (China). KOD-plus mutagenesis kits were obtained from TOYOBO (Japan). T4 DNA ligase and restriction endonucleases were obtained from Thermo Scientific (Pittsburgh, PA, USA). Phusion high-fidelity DNA polymerase was purchased from New England Biolabs (Ipswich, MA, USA). Ni^2+^-NTA Superflow was purchased from Qiagen Inc. (Germany). Polyethyleneimine cellulose plates were purchased from Merck (Germany). Pyrophosphatase (PPiase) was obtained from Roche Applied Science (China). The dNTP mixture was obtained from TaKaRa (Japan). Oligonucleotide primers were synthesized by Invitrogen (China). *Escherichia coli* BL21 (DE3) cells were purchased from Stratagene (Santa Clara, CA, USA). A diploid yeast strain (BY4743-MST1^+/−^) was obtained from Thermo Scientific. Recombinant plasmid pET28a-*Sc*cytThrRS was constructed in our laboratory (19). *C. albicans* genome was a gift from Prof. Jiang-Ye Chen in our Institute. p425TEF was kept in our laboratory (19).

### Cloning and mutagenesis

The *MST1* gene encoding the *Sc*mtThrRS precursor was amplified from the *S. cerevisiae* genome and cloned into pET28a(+) between the *Nde*I and *Xho*I restriction sites. The gene fragment encoding the mature *Sc*mtThrRS without its mitochondrial targeting sequence (MTS) (Met^1^-Ser^31^) (27) was then subcloned into pET28a(+) using the *Nde*I and *Xho*I sites to generate pET28a(+)-*Sc*mtThrRS, from which the mature *Sc*mtThrRS was expressed. Construction of the gene encoding the chimeric *S. cerevisiae* cytoplasmic-mitochondrial ThrRS (CmThrRS) was performed in two steps. First, the gene encoding the N-terminal fragment of *Sc*cytThrRS (Met^1^-Gln^337^, including the N-extension, N1 and N2 domains), was amplified by polymerase chain reaction (PCR) using pET28a- *Sc*cytThrRS as a template and cleaved by *Nde*I and *Sac*I enzymes. Second, the DNA fragment encoding the aminoacylation and C-terminal domains of *Sc*mtThrRS (Phe^49^-Lys^462^) was similarly obtained by PCR using pET28a-*Sc*mtThrRS as a template and digested by *Sac*I and *Xho*I. The two fragments were then co-ligated into pET28a pre-cleaved by *Nde*I and *Xho*I to obtain pET28a-CmThrRS. For construction of the chimeric CmThrRS2 gene, a DNA fragment encoding Met^1^-His^616^ of *Sc*cyt*ThrRS* (including the N-extension, N1, N2 and aminoacylation domains) was amplified by PCR using pET28a- *Sc*cyt*ThrRS* as a template and cleaved by *Nde*I and *Sac*I; a second DNA fragment encoding the C-terminal domains of *Sc*mtThrRS (Gly^339^-Lys^462^) was obtained by PCR using pET28a-*Sc*mtThrRS as a template and digested by *Sac*I and *Xho*I. The two fragments were then co-ligated into pET28a (pre-cleaved by *Nde*I and *Xho*I) to generate pET28a-CmThrRS2. For subcloning into p425TEF (32), the two genes encoding CmThrRS and CmThrRS2 were PCR amplified, digested and inserted into the gap between the *Pst*I and *Xho*I of p425TEF to obtain p425TEF-CmThrRS and p425TEF-CmThrRS2.

Genes encoding the *Sc*mtThrRS precursor or mature *Sc*mtThrRS were inserted into the yeast expression vector p425TEF at the *Pst*I and *Xho*I sites, respectively, to produce the p425TEF-*Sc*mtThrRS precursor (with the MTS) or p425TEF-*Sc*mtThrRS (without the MTS). Genes encoding *Ec*ThrRS or *Sc*cyt*ThrRS* were amplified from the *E. coli* genome or pET28a(+)-*Sc*cyt*ThrRS*, respectively, and inserted into the site of *Hind*III and *Sal*I or *Pst*I and *Xho*I sites, respectively, in p425TEF. The gene encoding mature *Ca*mtThrRS (Ser^29^-Lys^455^) (27) was amplified from the *C. albicans* genome, digested by *Pst*I and *Xho*I and inserted into the complementary sites of p425TEF. Construction of p425TEF-hcThrRS (human cytoplasmic ThrRS) has been described in a previous report (19). The gene fragment encoding the MTS of *Sc*mtThrRS (Met^1^-Ser^31^) was inserted just upstream of the open reading frame (ORF) of *Ec*ThrRS, *Sc*cyt*ThrRS*, hcThrRS, mature human mitochondrial ThrRS (hmtThrRS, Leu^20^-Phe^718^, unpublished results), mature *Ca*mtThrRS, CmThrRS and CmThrRS2 to facilitate guided mitochondrial import of these exogenously expressed proteins. All constructs were confirmed by DNA sequencing. DNA swapping and mutation were carried out according to the procedures provided with KOD mutagenesis kits.

### Protein gene expression and purification

*E. coli* BL21 (DE3) was transformed with various constructs. A single colony of each of the transformants was chosen and cultured in 500 ml of 2× YT medium at 37°C. When the cells reached mid-log phase (A_600_ = 0.6), expression of the recombinant proteins was induced by the addition of 0.2 mM isopropyl-1-thio-β-D-galactopyranoside for 8 h at 22°C. Protein purification was performed according to a previously described method (33).

### tRNA gene cloning and transcription

tRNA^Thr^1 and tRNA^Thr^2 genes were inserted between the *Pst*I and *EcoR*I sites of pTrc99b downstream of an 5′ inserted T7 promoter. All tRNA sequences were confirmed by DNA sequencing. Detailed *in vitro* T7 run-off transcription of tRNA^Thr^1 and tRNA^Thr^2 was performed as described previously (34). The accepting capacity of tRNA^Thr^1 and tRNA^Thr^2 was 1156 and 1327 pmol/A_260_, respectively. All tRNA mutants were constructed based on the protocol provided with KOD mutagenesis kits and transcribed as tRNA^Thr^1 and tRNA^Thr^2.

### Enzymatic assays

ATP-PPi exchange measurement was carried out at 30°C in a reaction mixture containing 60 mM Tris-HCl (pH 8.5), 10 mM MgCl_2_, 5 mM DTT, 0.1 mg/ml BSA, 2.5 mM ATP, 2 mM tetrasodium [^32^P]pyrophosphate, 1 mM Thr or 300 mM non-cognate Ser and 200 nM *Sc*mtThrRS or CmThrRS. Aliquots of 15 μl were taken and quenched to 200 μl with a solution containing 2% activated charcoal, 3.5% HClO_4_ and 50 mM tetrasodium pyrophosphate at various time intervals. The solution was filtered through a Whatman GF/C filter, followed by washing with 20 ml of 10 mM tetrasodium pyrophosphate solution and 10 ml of 100% ethanol. The filters were dried and [^32^P]ATP was measured using a scintillation counter (Beckman Coulter).

Assays of aminoacylation activity of *Sc*mtThrRS or various ThrRSs were performed at 30°C in a reaction mixture containing 60 mM Tris-HCl (pH 8.5), 10 mM MgCl_2_, 5 mM DTT, 0.1 mg/ml BSA, 2.5 mM ATP, 114.2 μM [^14^C]Thr, 5 μM tRNA^Thr^ or its variants and 200 nM or various amounts of ThrRS. The mis-aminoacylation experiment was performed at 30°C in the presence of 60 mM Tris-HCl (pH 8.5), 10 mM MgCl_2_, 5 mM DTT, 0.1 mg/ml BSA, 2.5 mM ATP, 250 μM [^14^C]Ser, 10 μM tRNA^Thr^1 or tRNA^Thr^2 and 2 μM *Sc*mtThrRS. [^14^C]Ser-tRNA^Thr^2 was prepared with *Sc*cyt*ThrRS* -H151A/H155A (19). Post-transfer editing activity of ThrRSs was indicated by the hydrolytic rate of [^14^C]Ser-tRNA^Thr^ and was measured at 30°C in a reaction mixture containing 60 mM Tris-HCl (pH 7.5), 10 mM MgCl_2_, 5 mM DTT, 0.1 mg/ml BSA, 2 μM [^14^C]Ser-tRNA^Thr^2 and 200 nM *Sc*mtThrRS or CmThrRS or CmThrRS-H151A/H155A. Aliquots were taken and quenched on Whatman filter pads pre-soaked with 5% trichloroacetic acid (TCA) at various time intervals. The filters were washed three times for 15 min each in cold 5% TCA and then three times for 10 min each in 100% ethanol. Filters were dried and the radioactivity content of the precipitates was quantified using a scintillation counter (Beckman Coulter).

### AMP formation assay

The AMP formation assay (thin-layer chromatography (TLC)) was carried out at 30°C in a reaction mixture containing 60 mM Tris-HCl (pH 8.5), 10 mM MgCl_2_, 5 mM DTT, 0.1 mg/ml BSA, 10 U/ml pyrophosphatase (PPiase), 40 mM Ser (or 4 mM Thr), 3 mM [α-^32^P]ATP and 2 μM *Sc*mtThrRS or CmThrRS in the presence or absence of tRNA^Thr^1 or tRNA^Thr^2 or its mutants. Samples (1.5 μl) were quenched in 6 μl of 200 mM NaAc (pH 5.0). The quenched aliquots (1.5 μl of each sample) were spotted onto polyethyleneimine cellulose plates pre-washed with water. Separation of Ser-[α-^32^P]AMP, [α-^32^P]AMP and [α-^32^P]ATP was performed in 0.1 M NH_4_Ac and 5% acetic acid. The plates were visualized by phosphorimaging and the data were analyzed using Multi Gauge Version 3.0 software (FUJIFILM). Quantification of [α-^32^P]AMP was achieved by densitometry in comparison with [α-^32^P]ATP samples of known concentrations. The rates were obtained using only the initial time points, where the plot of [α-^32^P]AMP versus time was linear. The data were then fit to the following equation: *y* = *b* + *k_ss_t*, where *b* and *k* represent the burst amplitude and the steady-state rate, respectively. The observed reaction rate constants (*k*_obs_) were obtained by dividing the steady-state rate of the reaction by the total enzyme concentration.

### *Sc*Δ*MST1* complementation assay

For complementation assays, all genes of interest were recombined into the yeast expression vector, p425TEF as described previously. Plasmids were introduced into *Sc*Δ*MST1* using the lithium acetate (LiAc) procedure (35). Transformants were selected on SD/Ura^−^/Leu^−^ plates and a single clone was cultured in liquid SD/Leu^−^ medium. The culture was then diluted to a concentration equivalent to 1 OD_600_ and a 10-fold dilution of the yeast was plated onto yeast-extract peptone glycerol (YPG) or YPG/5-FOA (5-floroorotic acid) to induce the loss of the rescue plasmid (pRS426-*MST1*). Complementation was observed by comparing the growth rates of *Sc*Δ*MST1* expressing *Sc*mtThrRS or various ThrRSs on YPG and YPG/5-FOA plates. The DNA fragment encoding a His_6_-tag was added downstream of the gene encoding CmThrRS or CmThrRS2 for facilitating a comparison of the levels of the two proteins expressed from *Sc*Δ*MST1*.

## RESULTS

### U33a and G36 of tRNA^Thr^1 are critical nucleotides for aminoacylation by *Sc*mtThrRS

**Figure 2.**
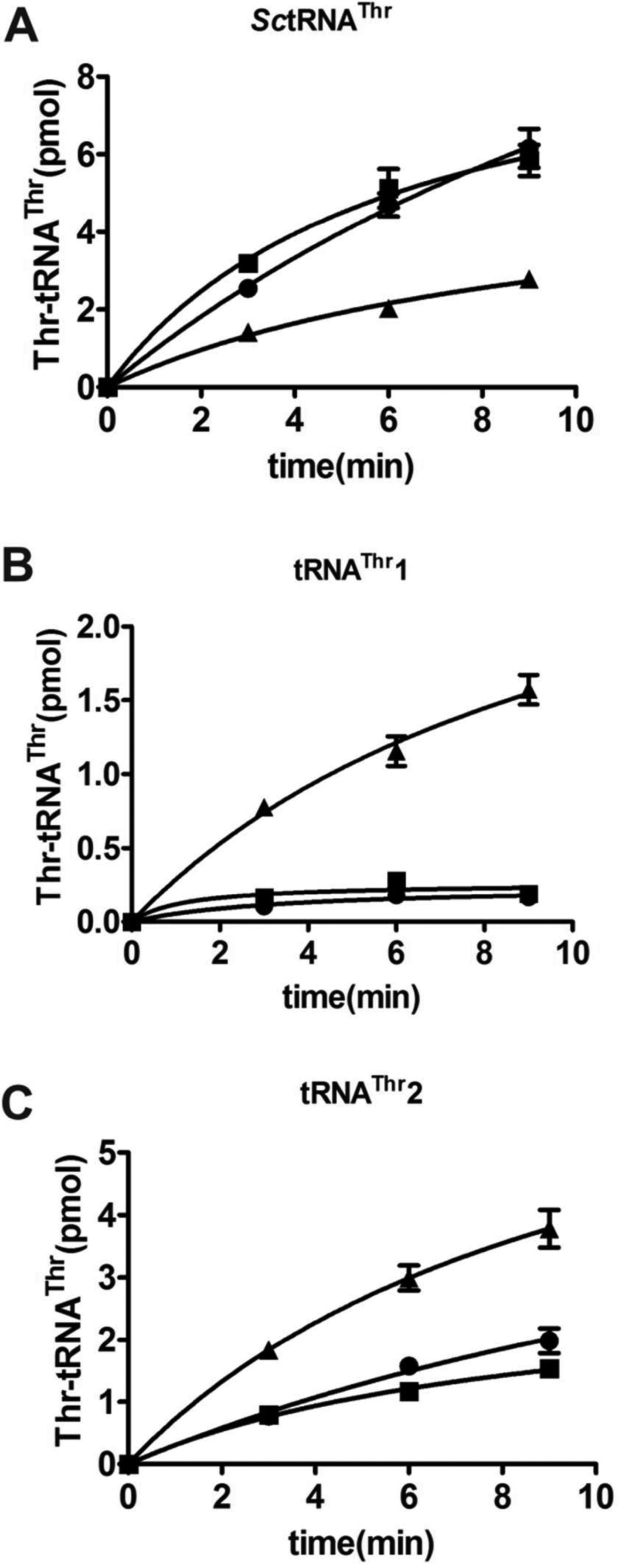
Cross-species aminoacylation of different tRNA^Thr^s by *Ec*ThrRS, *Sc*cyt*ThrRS S* and *Sc*mtThrRS. Aminoacylation time-course of *Sc*tRNA^Thr^ (A), tRNA^Thr^1 (B) and tRNA^Thr^2 (C) by *Ec*ThrRS (▪), *Sc*cyt*ThrRS* (•) and *Sc*mtThrRS (▴).

We initially investigated whether *E. coli* ThrRS (*Ec*ThrRS) and *S. cerevisiae* cytoplasmic ThrRS (*Sc*cyt*ThrRS*) were able to recognize the unusual tRNA^Thr^1 and whether *Sc*mtThrRS could aminoacylate other canonical forms of tRNA^Thr^, such as *S. cerevisiae* cytoplasmic tRNA^Thr^(AGU) (*Sc*tRNA^Thr^). Our data showed that all ThrRSs readily recognized canonical tRNA^Thr^s, including *Sc*tRNA^Thr^ and tRNA^Thr^2; however, only *Sc*mtThrRS was able to charge tRNA^Thr^1 (Figure 2). Therefore, *Sc*mtThrRS must use different tRNA^Thr^1-specific recognition elements and patterns (which are absent in *Ec*ThrRS and *Sc*cyt*ThrRS*) to aminoacylate tRNA^Thr^1.

**Figure 3.**
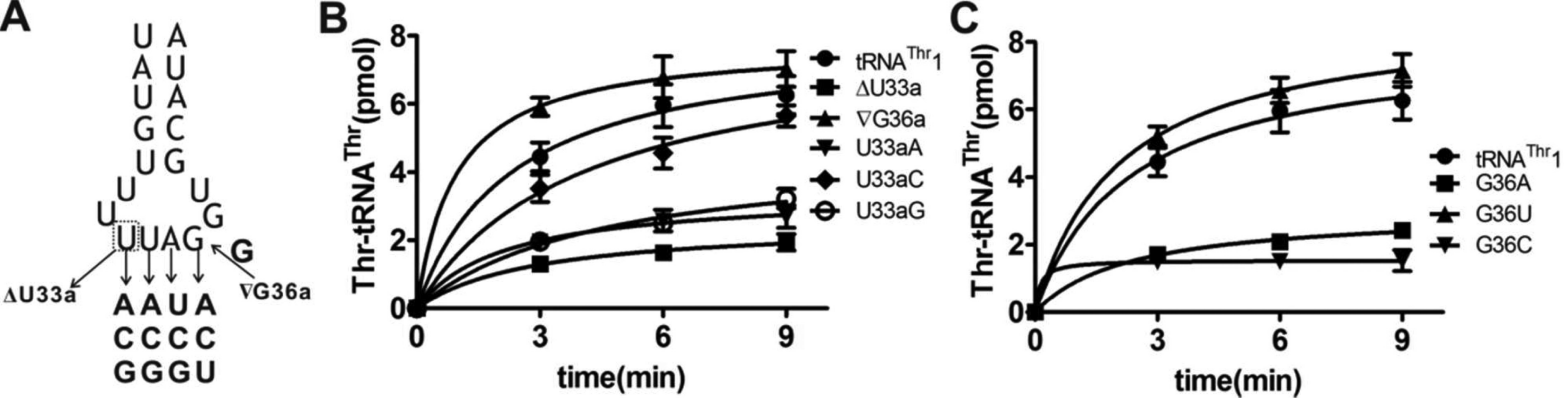
Contribution of the anticodon nucleotides of tRNA^Thr^1 to aminoacylation by *Sc*mtThrRS. (A) Scheme showing the anticodon stem-loop of tRNA^Thr^1 and the mutations studied. (B) Aminoacylation of native tRNA^Thr^1 (•) and its mutants, including ΔU33a (▪), ∇G36a (▴), U33aA (▾), U33aC (♦) and U33aG (○) by *Sc*mtThrRS. (C) Aminoacylation by *Sc*mtThrRS of native tRNA^Thr^1 (•) and mutated derivatives, including G36A (▪), G36U (▴) and G36C (▾).

The most striking feature of the unusual tRNA^Thr^1 is the enlarged anticodon loop. We deleted U33a or inserted an additional G36a in the anticodon loop to either reduce or further enlarge the size of the loop, thus, obtaining ΔU33a or ∇G36a (Figure 3A). Consistent with data from others (29), the size reduction decreased the rate of Thr acceptance to ∼70%, suggesting that the 8-nucleotide size of the anticodon loop plays a role in regulating tRNA charging. In contrast, aminoacylation of ∇G36a showed that size enlargement from eight to nine nucleotides slightly increased aminoacylation of the tRNA (Figure 3B).

Next, we examined if a U residue at position 33a was critical for a functional enlarged 8-nucleotide anticodon loop. We changed U33a to A, C or G to obtain U33aA, U33aC, U33aG mutants (Figure 3A). Aminoacylation assays showed that, compared to wild-type tRNA^Thr^1, the U33aC mutation slightly decreased accepting activity, whereas both the U33aA and U33aG mutations decreased the activity considerably (Figure 3B). These data showed that a pyrimidine (U or C) at position 33 was more suitable than a purine nucleotide (A or G). A pyrimidine nucleotide might directly interact with *Sc*mtThrRS or alternatively, contribute to anticodon loop plasticity during its interaction with the synthetase.

Finally, we mutated each of U34, A35 or G36 of tRNA^Thr^1 to the three other nucleotides to obtain U34A, U34C, U34G, A35U, A35C, A35G, G36A, G36C and G36U, respectively (Figure 3A). Charging assays showed that mutations at position 34 or 35 had little effect on aminoacylation (data not shown). In contrast, at position 36, the two mutants G36A and G36C showed a significant reduction in aminoacylation, indicating that G36 is an important determinant of *Sc*mtThrRS charging. The third nucleotide, G36U, displayed intact aminoacylation properties (Figure 3C). We further calculated aminoacylation kinetics of *Sc*mtThrRS for all the mutants derived from U33a and G36. The data showed that only U33aC and G36U displayed nearly full aminoacylation activity (99.8% and 76%, respectively); however, activity of other mutants decreased to only about 10% of that of wild-type tRNA^Thr^1 (Table 1). Our data were consistent with results of Ling *et al.*, who deleted the U33a or simultaneously mutated A35 and G36 to G and U (obtaining A35G/G36U mutant) and revealed that the inserted U33a played a crucial role in aminoacylation (29).

**Table 1.** Aminoacylation kinetics of *Sc*mtThrRS for various tRNA^Thr^1 mutants derived from U33a or G36

tRNA	*k*_cat_ (min^−1^)	*K*_m_ (μM)	*k*_cat_/*K*_m_ (min^−1^ μM^−1^)	Relative (%)
tRNA^Thr^1	2.03 ± 0.32	0.45 ± 0.05	4.51	100
ΔU33a	0.71 ± 0.10	2.07 ± 0.25	0.34	7.5
U33aA	0.82 ± 0.18	1.91 ± 0.24	0.43	9.5
U33aC	1.98 ± 0.17	0.44 ± 0.04	4.50	99.8
U33aG	0.89 ± 0.14	2.20 ± 0.31	0.40	8.9
G36A	0.73 ± 0.16	1.87 ± 0.22	0.39	8.6
G36C	0.69 ± 0.13	1.56 ± 0.19	0.44	9.8
G36U	2.06 ± 0.25	0.60 ± 0.07	3.43	76.1

The results are the average of three independent repeats with standard deviations indicated. The *k*_cat_/*K*_m_ values are relative to tRNA^Thr^1.

### *Sc*mtThrRS has isoacceptor-specific tRNA-dependent pre-transfer editing activity

**Figure 4.**
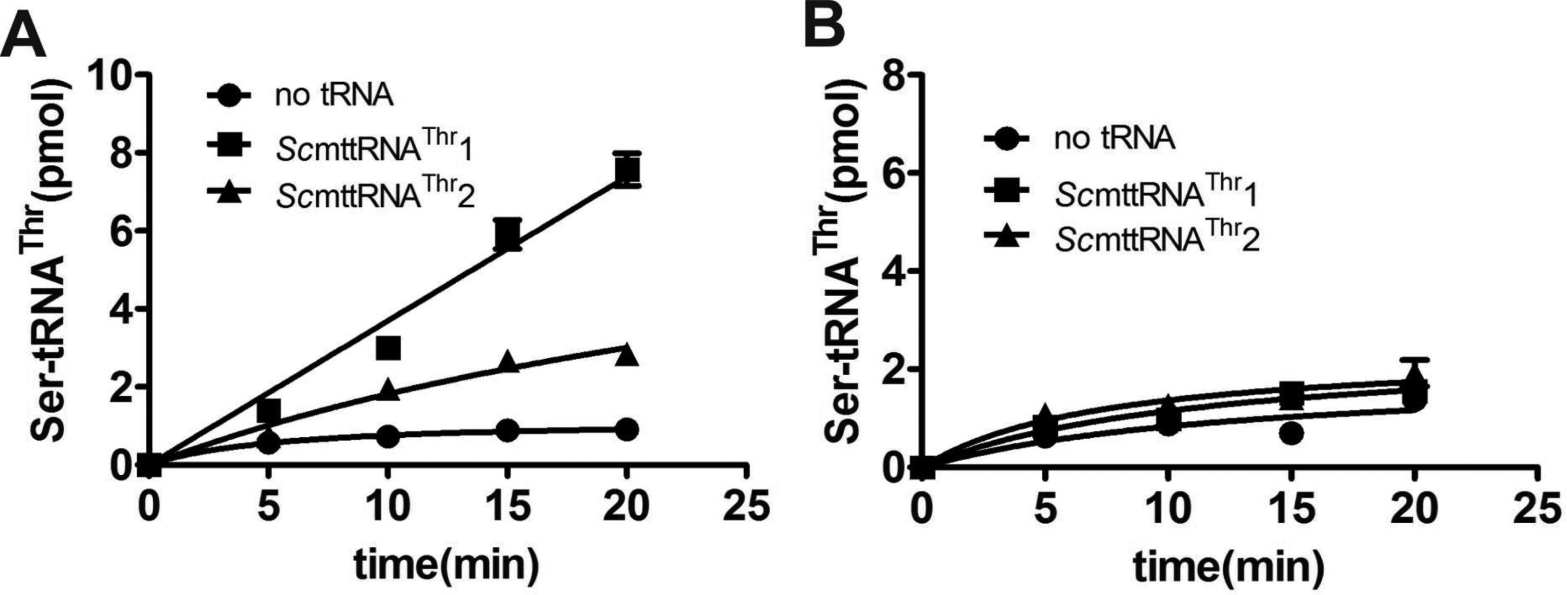
Mis-charging of mitochondrial tRNA^Thr^s by *Sc*mtThrRS and CmThrRS. Mis-charging time-course of *Sc*mttRNA^Thr^1 (▪) and *Sc*mttRNA^Thr^2 (▴) with non-cognate Ser catalyzed by *Sc*mtThrRS (A) and CmThrRS (B). Mis-charging reaction in the absence of tRNA (•) was performed as a control for either enzyme.

**Figure 5.**
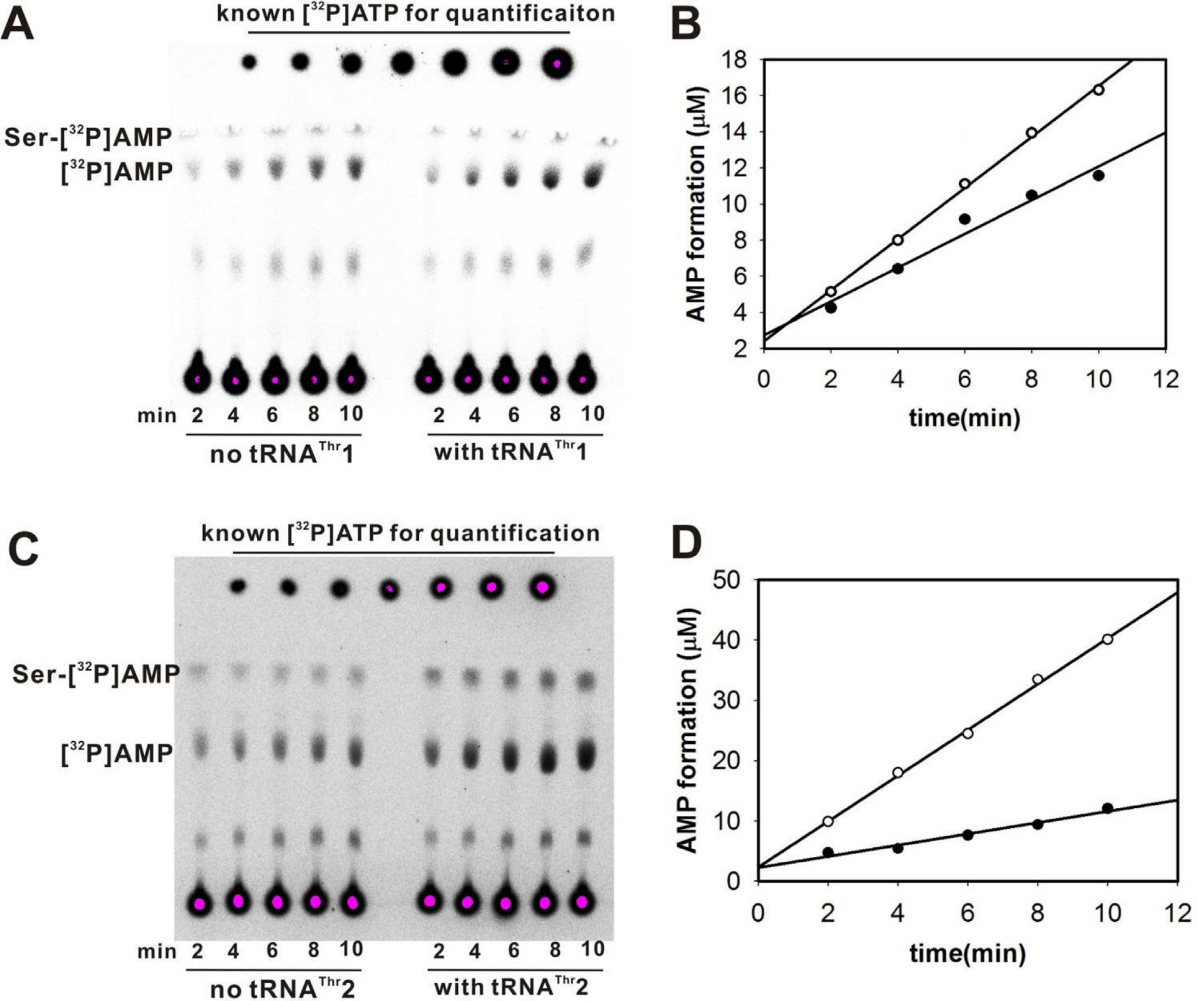
Isoacceptor-specific editing by *Sc*mtThrRS. (A) Representative TLC plate of the editing assay performed in the presence of Ser and *Sc*mtThrRS in the absence or presence of tRNA^Thr^1. [^32^P]AMP and Ser-[^32^P]AMP are indicated. (B) Graphic representation of AMP formation without (•) or with (○) tRNA^Thr^1 as shown in (A). (C) Representative AMP formation assay with Ser catalyzed by *Sc*mtThrRS in the absence or presence of tRNA^Thr^2. (D) Graphic representation of AMP formation without (•) or with (○) tRNA^Thr^2 as shown in (C). Known amounts of [α-^32^P]ATP were serially diluted and spotted onto the TLC plate in (A) and (C) after separation for quantification.

Recently, it was shown that *Sc*mtThrRS catalyzes the mis-activation of non-cognate Ser and uses pre-transfer editing to hydrolyze Ser-AMP (31). However, despite the presence of pre-transfer editing activity against Ser, *Sc*mtThrRS still formed Ser-tRNA^Thr^ *in vitro* (31) indicating that the editing activity was not sufficient to prevent Ser mis-charging. Here, we performed mis-charging assays with non-cognate Ser and confirmed that both tRNA^Thr^1 and tRNA^Thr^2 were mis-charged by Ser with a higher rate for tRNA^Thr^1 (with *k*_obs_ of [(0.36 ± 0.05) × 10^−3^ s^−1^]) compared to tRNA^Thr^2 (with *k*_obs_ of [(0.11 ± 0.02) × 10^−3^ s^−1^]) (Figure 4A) (*k*_obs_ value was calculated with the same equation with AMP formation as described in the Materials and Methods). Such a preference for tRNA^Thr^1 has already been reported (31) showing that *Sc*mtThrRS is an error-prone tRNA synthetase, at least *in vitro*. Here, we evaluated the tRNA-dependent pre-transfer editing activity of *Sc*mtThrRS since, theoretically, tRNA^Thr^ isoacceptors might also bind the enzyme before the hydrolysis or release of Ser-AMP. Therefore, we performed editing assays in the presence of the non-cognate amino acid Ser using TLC-based AMP formation from [α-^32^P]ATP. Compared to the traditional [α-^32^P]ATP consumption assay, the AMP formation assay allows the simultaneous, direct separation and measurement of [α-^32^P]AMP and aminoacyl-[α-^32^P]AMP (17). We performed AMP formation assays with *Sc*mtThrRS in the presence of either tRNA^Thr^1 or tRNA^Thr^2. The results showed that tRNA^Thr^1 stimulated the tRNA-dependent pre-transfer editing activity of *Sc*mtThrRS only slightly, with *k*_obs_ of (11.78 ± 1.54) × 10^−3^ s^−1^ (with tRNA^Thr^1) as compared to a *k*_obs_ of (7.78 ± 1.12) × 10^−3^ s^−1^ without tRNA^Thr^1 (Table 2). These data suggest that *Sc*mtThrRS has little tRNA^Thr^1-dependent pre-transfer editing activity (Figure 5A and B). In contrast, tRNA^Thr^2 stimulated greater tRNA-dependent pre-transfer editing of *Sc*mtThrRS with a *k*_obs_ of (30.39 ± 2.63) × 10^−3^ s^−1^ (Figure 5C and D) (Table 2). Compared to the rate of formation of Ser-tRNAs^Thr^ [(0.36 ± 0.05) and (0.11 ± 0.02) × 10^−3^ s^−1^] for tRNA^Thr^1 and tRNA^Thr^2, respectively, the AMP formation rates are much higher indicating that ATP was exhausted during the editing assay. We further performed Thr-included AMP formation assays in the absence or presence of either tRNA^Thr^1 or tRNA^Thr^2. Data showed that, without any tRNA, the *k*_obs_ of AMP formation with Thr [(2.65 ± 0.32) × 10^−3^ s^−1^] was significantly lower than that with Ser [(7.78 ± 1.12) × 10^−3^ s^−1^]. Similarly, the *k*_obs_ value of AMP formation with Thr in the presence of either tRNA^Thr^1 [(3.25 ± 0.30) × 10^−3^ s^−1^] or with tRNA^Thr^2 [(5.24 ± 0.86) × 10^−3^ s^−1^] was obviously lower than that with Ser in the presence of tRNA^Thr^1 [(11.78 ± 1.54) × 10^−3^ s^−1^] or tRNA^Thr^2 [(30.39 ± 2.63) × 10^−3^ s^−1^] (Table 2). Therefore, these data indicated that Ser-induced AMP formation was the result of an editing reaction.

**Table 2.** Observed rate constants of AMP formation by *Sc*mtThrRS with Ser in the presence or absence of tRNA^Thr^s

tRNA	*k*_obs_ (× 10^−3^) (s^−1^)^a^
No tRNA	7.78 ± 1.12
tRNA^Thr^1	11.78 ± 1.54
tRNA^Thr^2	30.39 ± 2.63

^a^The results are the average of three independent repeats with standard deviations indicated.

### Role of anticodon of tRNA^Thr^2 in tRNA-dependent pre-transfer editing by *Sc*mtThrRS

In a previous study, we showed that the conserved G35 and U36 are key determinants of editing by *Sc*cyt*ThrRS*, whereas the discriminator base A73 is of little importance in editing (19). To check whether this editing recognition mode was conserved in mitochondrial tRNA^Thr^2, we mutated G35, U36 and A73 to C, obtaining G35C, U36C and A73C, respectively. The aminoacylation of all the mutants was severely impacted (data not shown), consistent with their function as recognition elements in the *Ec*ThrRS and *Sc*cyt*ThrRS* systems (19). We then used these mutants to measure tRNA-dependent AMP formation by *Sc*mtThrRS in the presence of non-cognate Ser. We found that the A73C mutant displayed nearly full efficiency [*k*_obs_ = (29.85 ± 3.07) × 10^−3^ s^−1^] compared to wild-type tRNA^Thr^2 [*k*_obs_ = (30.39 ± 2.63) × 10^−3^ s^−1^]. In sharp contrast, the *k*_obs_ values of G35C [(11.30 ± 1.76) × 10^−3^ s^−1^] and U36C [(4.85 ± 0.64) × 10^−3^ s^−1^] fell to a level close to that of tRNA-independent pre-transfer editing [(7.78 ± 1.12) × 10^−3^ s^−1^], suggesting that the tRNA mutants were not able to stimulate pre-transfer editing (Table 3). In summary, aminoacylation-impaired A73C stimulated a similar level of editing compared with wild-type tRNA^Thr^2; thus, A73 is only critical for the synthetic activity, while G35 and U36 play a role in both the aminoacylation and editing activities.

**Table 3.** Observed rate constants of AMP formation by *Sc*mtThrRS with non-cognate Ser in the presence of tRNA^Thr^2 or mutated derivatives

tRNA	*k*_obs_ (× 10^−3^) (s^−1^)^b^	Relative *k*_obs_ (%)^a^
tRNA^Thr^2	30.39 ± 2.63	100
G35C	11.30 ± 1.76	37
U36C	4.85 ± 0.64	16
A73C	29.85 ± 3.07	97

^a^The *k*_obs_ values are relative to that of tRNA^Thr^2.

^b^The results are the average of three independent repeats with standard deviations indicated.

### tRNA^Thr^1 stimulates pre-transfer editing in the presence of an editing domain

**Figure 6.**
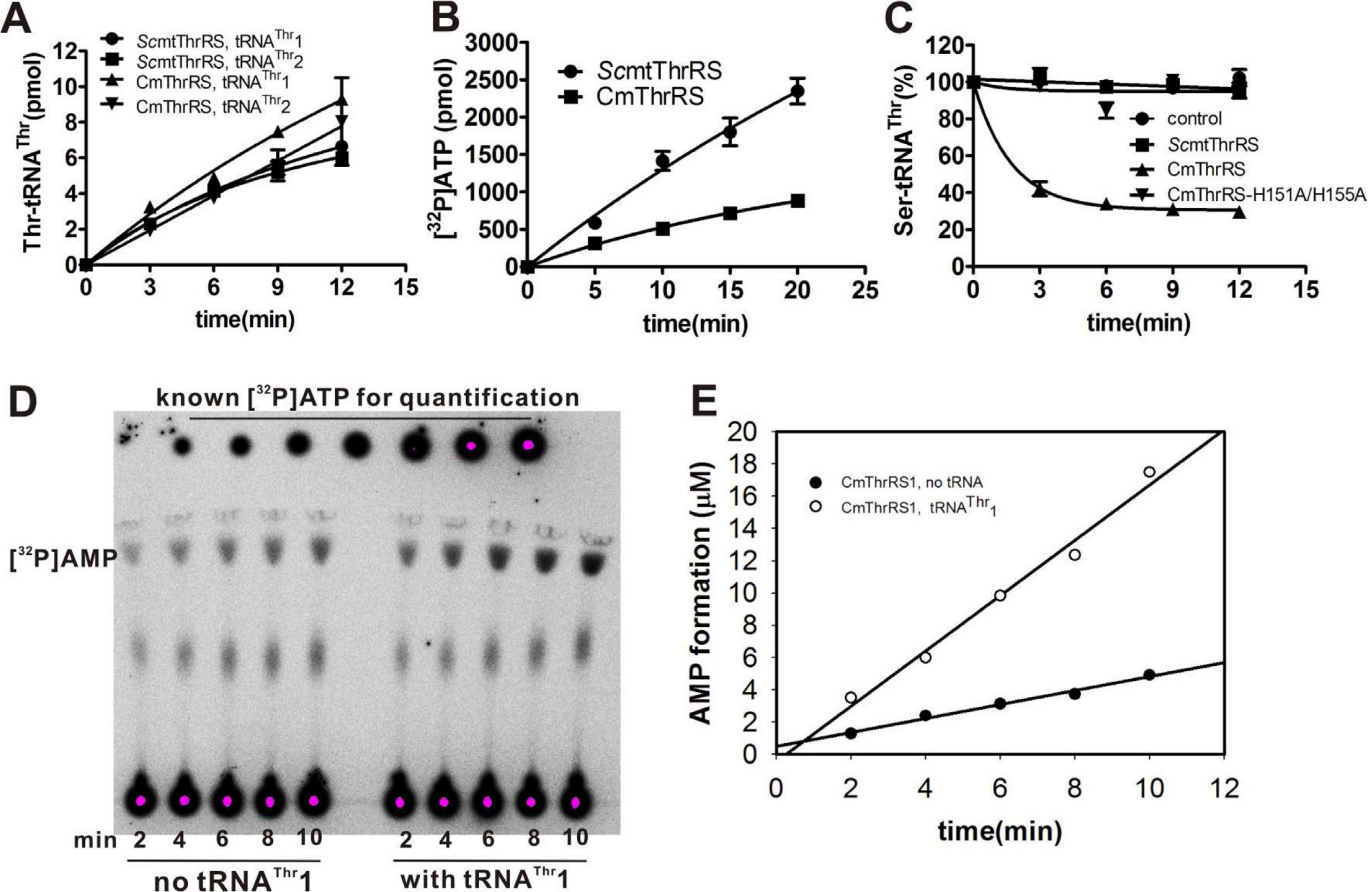
Fusion of the editing domain to *Sc*mtThrRS restored its tRNA^Thr^1-dependent pre-transfer editing capacity. (A) Aminoacylation of tRNA^Thr^1 or tRNA^Thr^2 by either *Sc*mtThrRS (• for tRNA^Thr^1 and ▪ for tRNA^Thr^2) or CmThrRS (▴ for tRNA^Thr^1 and ▾ for tRNA^Thr^2). (B) ATP-PPi exchange assay showing Thr activation catalyzed by *Sc*mtThrRS (•) and CmThrRS (▪). (C) Deacylation of Ser-tRNA^Thr^2 by *Sc*mtThrRS (▪), CmThrRS (▴) and CmThrRS-H151A/H155A (▾). Spontaneous hydrolysis of mis-charged tRNA (control) was also carried out in the absence of enzyme (•). (D) Representative TLC plate showing AMP formation catalyzed by CmThrRS in the absence or presence of tRNA^Thr^1. (E) Graphic representation of AMP formation without (•) or with (○) tRNA^Thr^1 detected in (C). A series of known amounts of [^32^P]ATP were loaded for quantification.

We previously showed that the N2 editing domain of *Sc*cyt*ThrRS* contributes to both the aminoacylation and editing activities (19). Therefore, as tRNA^Thr^1 was unable to stimulate tRNA-dependent pre-transfer editing, we checked if the absence of the editing domain in *Sc*mtThrRS could explain this incapacity. To address this question, we first added the complete N-terminal domain of the cytosolic *Sc*cyt*ThrRS*, including the N1 and N2 editing domains (Met^1^-Gln^337^), to *Sc*mtThrRS. This chimeric enzyme, designated CmThrRS (cytoplasmic-mitochondrial ThrRS), showed some remarkable catalytic features. First, CmThrRS exhibited intact aminoacylation activity for both tRNA^Thr^1 and tRNA^Thr^2 substrates (Figure 6A), despite a decrease in the Thr activation rate to 30% of the wild-type level (Figure 6B). Second, we observed that the added editing domain in CmThrRS induced recovery of the post-transfer editing activity as shown by deacylation of Ser-tRNA^Thr^ (Figure 6C), thus, CmThrRS accumulated neither Ser-tRNA^Thr^1 nor Ser-tRNA^Thr^2 (Figure 4B). Third, AMP formation was measured in the absence or presence of tRNA^Thr^1 in order to clarify whether tRNA^Thr^1 was able to stimulate pre-transfer editing by CmThrRS. In the presence of non-cognate Ser, AMP formation was induced significantly in the presence of tRNA^Thr^1 with a *k*_obs_ of (14.30 ± 2.31) × 10^−3^ s^−1^, which was almost 4-fold higher than *k*_obs_ in the absence of tRNA^Thr^1 (3.59 ± 0.74) × 10^−3^ s^−1^ (Figure 6D and E). Similarly, tRNA^Thr^2 stimulated AMP formation by CmThrRS with an even higher *k*_obs_ of (20.86 ± 2.10) × 10^−3^ s^−1^ (Table 4); however, this value was still lower than that of the original *Sc*mtThrRS [(30.39 ± 2.63) × 10^−3^ s^−1^, Table 3] (here, the AMP formation of CmThrRS with tRNA^Thr^1 or tRNA^Thr^2 included both pre-transfer editing and post-transfer editing since CmThrRS harbors an active editing domain). These data revealed that tRNA^Thr^1 has the intrinsic capacity to stimulate editing, but requires the presence of an additional editing domain in *Sc*mtThrRS. We have demonstrated that His^151^ and His^155^ in the editing domain of *Sc*cyt*ThrRS* are responsible for the post-transfer editing reaction but not for pre-transfer editing (19). To verify that the increased AMP formation rate was due to pre-transfer and not post-transfer activity, we mutated residues His^151^ and His^155^ of the CmThrRS derived from *Sc*cyt*ThrRS* to Ala residues to produce a mutant CmThrRS-H151A/H155A. As expected, the double mutant was deficient in post-transfer editing activity and could not deacylate preformed Ser-tRNA^Thr^ (Figure 6C) but it could still catalyze tRNA^Thr^1- or tRNA^Thr^2-dependent pre-transfer editing with *k*_obs_ values of (12.75 ± 2.21) × 10^−3^ s^−1^ or (14.20 ± 2.67) × 10^−3^ s^−1^, respectively (Table 4) (here, the AMP formation of CmThrRS-H151A/H155A with tRNAs included only pre-transfer editing). In combination, these data suggested that tRNA^Thr^1 has the intrinsic capacity to stimulate pre-transfer editing, but requires the presence of a classical editing domain to express this activity.

**Table 4.** Observed rate constants of AMP formation by CmThrRS or CmThrRS-H151A/H155A with non-cognate Ser in the presence of the two tRNA^Thr^ isoacceptors

Enzyme	tRNA	*k*_obs_ (× 10^−3^) (s^−1^)^a^
CmThrRS	No tRNA	3.59 ± 0.74
	tRNA^Thr^1	14.30 ± 2.31
	tRNA^Thr^2	20.86 ± 2.10
CmThrRS-H151A/H155A	tRNA^Thr^1	12.75 ± 2.21
	tRNA^Thr^2	14.20 ± 2.67

^a^The results are the average of three independent repeats with standard deviations indicated.

### Role of the tRNA^Thr^1 anticodon in editing by CmThrRS

We previously showed that the anticodon nucleotides of *Sc*tRNA^Thr^ or tRNA^Thr^2 are critical for the pre-transfer editing activity of *Sc*cyt*ThrRS* or *Sc*mtThrRS (Table 3) (19). Here we showed that, despite its extended size in anticodon loop, the tRNA^Thr^1 stimulated the tRNA-dependent editing activity of CmThrRS in the presence of non-cognate Ser. Therefore, to explore the plasticity of the anticodon loop, especially of the ^33a^UUAG^36^ tetranucleotide of tRNA^Thr^1 during pre-transfer editing stimulation, we carried out AMP formation assays using the chimeric enzyme CmThrRS in the presence of our constructed ΔU33a, ∇G36a, U33aA, U33aC, U33aG, U34A, U34C, U34G, A35U, A35C, A35G, G36A, G36C or G36U forms of tRNA^Thr^1. Results from the editing stimulation assays could be classified into three major categories. Nine of the 14 mutants (∇G36a, U33aC, U33aG, U34A, U34C, U34G, A35U, A35G and G36C) did not have an obvious effect on editing by CmThrRS compared with native tRNA^Thr^1 (Table 5). Three mutants (ΔU33a, A35C and G36A) exhibited decreased (by ∼50%) editing stimulation with *k*_obs_ values of (7.92 ± 0.94) × 10^−3^ s^−1^, (7.96 ± 0.87) × 10^−3^ s^−1^ and (8.35 ± 1.32) × 10^−3^ s^−1^, respectively. Finally, a third category, comprising two mutants (U33aA and G36U) exhibited *k*_obs_ values of (4.51 ± 0.56) × 10^−3^ s^−1^ and (3.56 ± 0.48) × 10^−3^ s^−1^, respectively, comparable with that in absence of tRNA (3.59 ± 0.74) × 10^−3^ s^−1^, which demonstrated the failure of these two mutants to induce significant tRNA-dependent editing (Table 5). These data indicated that the editing activity of CmThrRS is sensitive to anticodon loop size reduction and to specific mutations of nucleotides 33a, 35 and 36 of tRNA^Thr^1. In particular, G36U, which exhibited equivalent aminoacylation activity compared with wild-type tRNA^Thr^1 (Figure 3C and Table 1), failed to stimulate any editing, further suggesting that Ser-induced AMP formation was the result of editing but not aminoacylation activity.

**Table 5.** Observed rate constants of AMP formation by CmThrRS with non-cognate Ser in the presence of tRNA^Thr^1 or mutated derivatives

tRNA	*k*_obs_ (× 10^−3^) (s^−1^)^b^	Relative *k*_obs_ (%)^a^
No tRNA	3.59 ± 0.74	25
tRNA^Thr^1	14.30 ± 2.31	100
ΔU33a	7.92 ± 0.94	55
∇G36a	15.91 ± 2.79	111
**U33aA**	**4.51** ± **0.56**	**32**
U33aC	14.47 ± 1.89	101
U33aG	11.98 ± 2.01	84
U34A	11.01 ± 1.35	77
U34C	9.82 ± 1.86	69
U34G	9.02 ± 1.12	63
A35U	12.39 ± 2.75	87
A35C	7.96 ± 0.87	56
A35G	14.76 ± 2.16	103
G36A	8.35 ± 1.32	58
G36C	10.40 ± 1.88	73
**G36U**	**3.56** ± **0.48**	**25**

^a^The *k*_obs_ values are relative to that of tRNA^Thr^1.

^b^The results are the average of three independent repeats with standard deviations indicated.

Values of U33aA and G36U mutants, which are significantly reduced, are shown in bold.

### The *MST1* gene knockout strain, *Sc*Δ*MST1*, reveals that aminoacylation and tRNA binding domains co-evolved to acquire tRNA^Thr^1 recognition capability

Both the primary and tertiary structures of *Sc*mtThrRS and other ThrRSs (such as *Ec*ThrRS) are highly similar (Supplementary Figure S1); however, only *Sc*mtThrRS has the capacity to acylate tRNA^Thr^1, indicating that tRNA^Thr^1-specific recognition elements are highly secluded and difficult to identify. Indeed, extensive *in vitro* structure-guided single-point mutagenesis in the C-terminal domain of *Sc*mtThrRS provided some insights but did not reveal critical residues specific only for tRNA^Thr^1 (29). Therefore, we constructed a yeast *MST1* gene knockout strain to establish a genetic complementation assay to investigate the *in vivo* complementation capacity of other ThrRSs and to provide insights into tRNA^Thr^1 recognition.

We purchased a diploid yeast strain (BY4743-MST1^+/−^) from Thermo Scientific, exhibiting one wild-type copy of *MST1*, while the other copy was replaced by a kanamycin gene (36). Strain BY4743-MST1^+/−^ was transformed with the rescue plasmid [pRS426-*MST1*, (pRS426: *MST1*^+^, Ura^+^)] and transformants were cultured on Dropout minimal media minus uracil (SD/Ura^−^). Ura^+^ colonies were selected and sporulation was induced. Tetrads were dissected and separated on YPG plates. YPG respiratory and SD/Ura^−^ media supported growth of the haploid *MST1*-knockout (*Sc*Δ*MST1*); however, the strain did not survive on YPG plates supplemented with 5-FOA, the toxic product of which, 5-flurouracil, excluded the rescue plasmid (Supplementary Figure S2A). This result showed that *MST1* is an essential gene for respiratory metabolism. We also confirmed *MST1* knockout using a PCR-based method (Supplementary Figure S2B and C).

**Figure 7.**
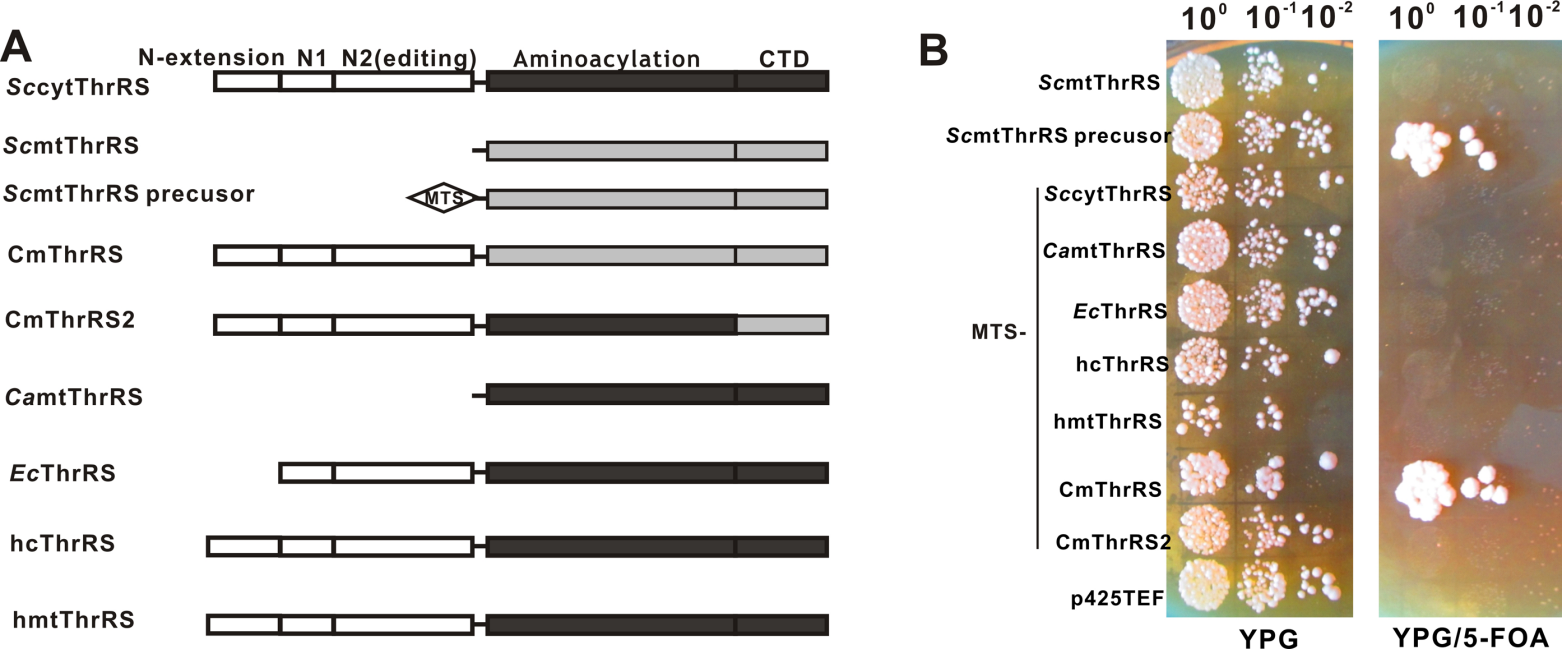
Complementation assay of the yeast knockout strain *Sc*ΔMST1 by different ThrRS genes and chimeric constructs. (A) Scheme showing domain composition of the various ThrRSs tested. N-terminal domains (including N-extension, N1 and N2 editing domains) are colored white; aminoacylation and CTDs of *Sc*mtThrRS or of other ThrRSs are colored gray or black, respectively. The MTS of *Sc*mtThrRS precursor is indicated by a diamond. (B) Shuffle assay performed under respiratory conditions without (YGP) or with 5-FOA (YPG/5-FOA) to induce loss of the rescue plasmid. The p425TEF empty vector was introduced as a negative control. Sequence encoding the functional MTS of *Sc*mtThrRS precursor was added before the ORF of all ThrRSs (including natural cytoplasmic or mature mitochondrial ThrRSs).

We then tested several ThrRSs originating from different organisms in the *Sc*Δ*MST1* strain (Figure 7A). The genes of these proteins were recombined into the yeast expression vector p425TEF. The entire ORF of the *MST1* precursor was first cloned as well as the protein deprived of its MTS. These constructs, together with the p425TEF empty vector were introduced into *Sc*Δ*MST1* and transformants were grown on YPG and YPG/5-FOA plates. The 5-FOA supplemented respiratory-medium supported growth of clones harboring the gene for the *Sc*mtThrRS precursor only, confirming that the MTS is a critical element for targeting exogenously expressed mature *Sc*mtThrRS into the mitochondrion (Figure 7B).

Subsequently, we tested several other natural ThrRSs, including cytosolic *Sc*cyt*ThrRS*, *Ec*ThrRS, mature *Ca*mThrRS (Ser^29^-Lys^455^), hcThrRS and mature hmtThrRS (Leu^20^-Phe^718^) (Figure 7A). Genes encoding these ThrRSs were ligated downstream of the sequence encoding the MTS. Shuffle assays on 5-FOA-medium showed that all these natural ThrRSs were unable to rescue respiratory deficiency (Figure 7B). This raises the question of whether the origin of these deficiencies was the lack of tRNA^Thr^1 aminoacylation or of mitochondrial import. From our present aminoacylation studies (Figure 2) and reports by others (27), we know that *Ec*ThrRS, *Sc*cyt*ThrRS* and *Ca*mtThrRS readily charge tRNA^Thr^2 but not tRNA^Thr^1 *in vitro*. Here, the shuffle assay confirmed that aminoacylation did not occur *in vivo* either. On the other hand, to further test the mitochondrial import capacity of the MTS with an exogenous ThrRS, we added the MTS upstream of the chimeric CmThrRS that was able to aminoacylate tRNA^Thr^1 to form MTS-CmThrRS (Figure 6A). The knockout strain was growth-capable under respiratory conditions, showing that MTS-CmThrRS complemented the yeast strain (Figure 7B). These data confirmed that the MTS efficiently directed import of the exogenous ThrRS sequence and strongly implied that the natural enzymes were inefficient in aminoacylating tRNA^Thr^1 *in vivo*.

**Figure 8.**
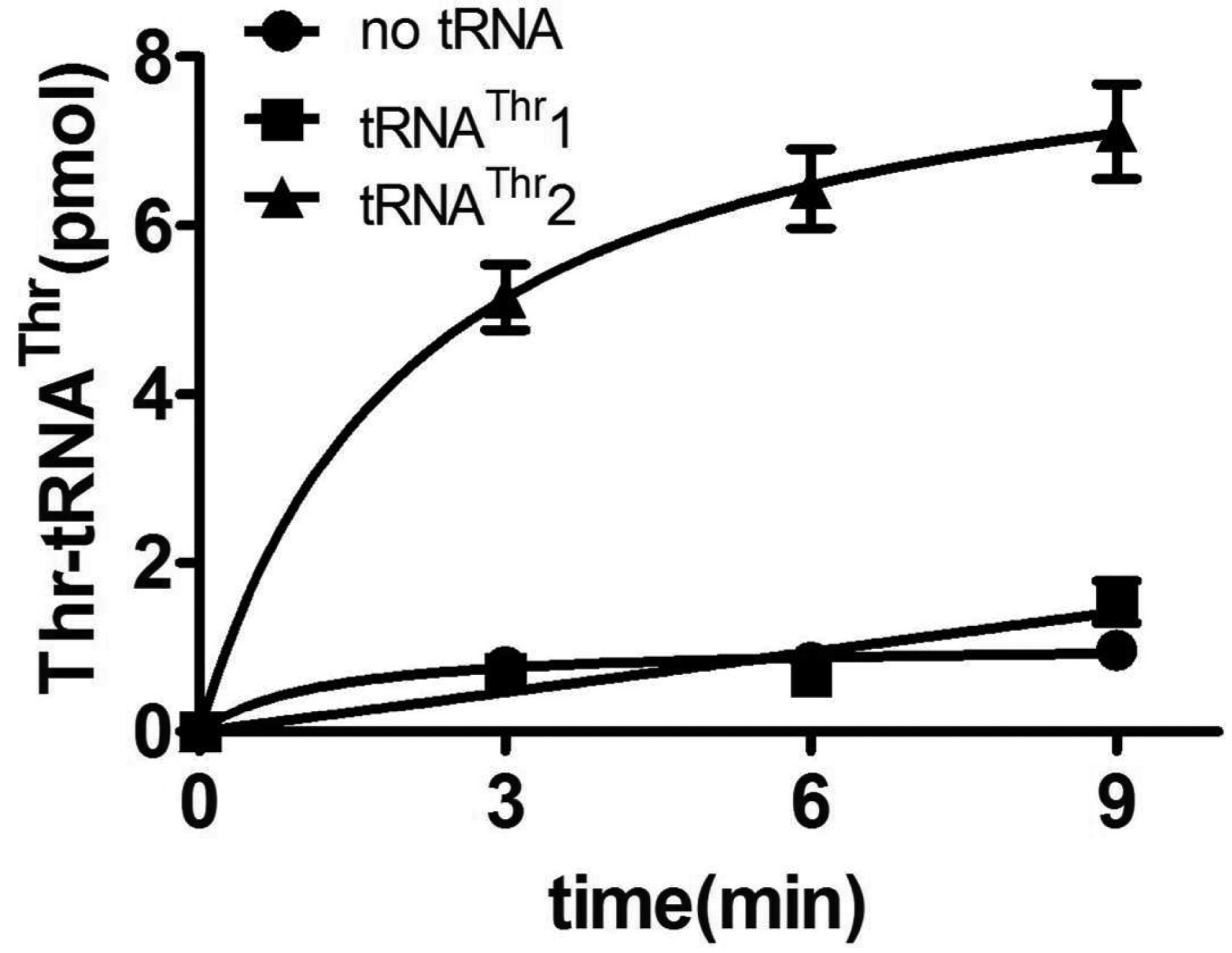
Aminoacylation activity of CmThrRS2. Aminoacylation of tRNA^Thr^1(▪) or tRNA^Thr^2 (▴) by CmThrRS2. Reaction without tRNA addition (•) was performed as a negative control.

Since only the MTS-CmThrRS and *Sc*mtThrRS precursor, both harboring the aminoacylation and C-terminal domains of mitochondrial origin, were able to complement the yeast strain, we speculated that the presence of the mitochondrial C-terminal domain was responsible for *in vivo* aminoacylation of tRNA^Thr^1. To address this question, we replaced the C-terminal domain of *Sc*cyt*ThrRS* with its counterpart derived from *Sc*mtThrRS to generate CmThrRS2 (Figure 7A). Therefore, CmThrRS2 and CmThrRS differed only in the aminoacylation domains, which were of cytoplasmic and mitochondrial origin, respectively (Figure 7A). After fusion with the MTS, MTS-CmThrRS2 was found to be unable to support mitochondrial protein synthesis *in vivo* despite comparable levels of CmThrRS and CmThrRS2 protein in *Sc*Δ*MST1* (Supplementary Figure S3). *In vitro* aminoacylation data also confirmed that CmThrRS2 charged tRNA^Thr^2 but not tRNA^Thr^1 (Figure 8). Therefore, our results indicated that only a ThrRS with both mitochondrial aminoacylation and C-terminal domains are capable of charging tRNA^Thr^1, suggesting that the two domains co-evolved to confer tRNA^Thr^1 aminoacylation activity.

## DISCUSSION

### Mitochondrial ThrRS deprived of the editing domain catalyzes tRNA-dependent pre-transfer editing

ThrRS is a class II synthetase with a unique modular structure containing three structural domains. The dimeric core consists mainly of the synthetic catalytic site and the C-terminal tRNA-anticodon binding domain. One extra domain on the N-terminal side of each monomer protrudes outside the core, forming the editing domains of the dimer (37). Such modular organization is conserved from bacteria (such as *E. coli*), to higher eukaryotes (such as humans) and in cytoplasmic and mitochondrial compartments. Among them, bacteria and eukaryotic cytoplasmic ThrRSs have been shown to predominantly use post-transfer editing reactions to prevent the synthesis of Ser-tRNA^Thr^ (19,30). In the archaeal kingdom, several ThrRSs (such as in *Pyrococcus abyssi*) use particular editing domains related to D-tyrosyl-tRNA^Tyr^ deacylases to hydrolyze Ser-tRNA^Thr^ (38,39). Other archaeal ThrRSs (such as in *Sulfolobus solfataricus*) are devoid of editing domains; however, editing of Ser-tRNA^Thr^ is maintained and catalyzed by an unrelated free-standing editing domain (40). All these studies underline the crucial importance of post-transfer editing as a quality control mechanism of the tRNA^Thr^ aminoacylation reaction. Despite this evidence, the N-terminal editing domains of mitochondrial ThrRSs from several yeast species (such as *S. cerevisiae*, *S. pombe* and *C. albicans*) have been lost and these enzymes are defective in post-transfer editing of Ser-tRNA^Thr^. It was previously shown that *S. cerevisiae* mitochondrial ThrRS (*Sc*mtThrRS) harbored a tRNA-independent pre-transfer editing activity for hydrolysis of Ser-AMP (31). In the present study, we showed that *Sc*mtThrRS also possesses a tRNA-dependent pre-transfer activity that is stimulated by tRNA^Thr^2 but not tRNA^Thr^1. Indeed, AMP formation of *Sc*mtThrRS at the presence of tRNA^Thr^2 was ∼4-fold compared with that of without tRNA; however, the unusual tRNA^Thr^1 only induced AMP production very modestly. A similar tRNA synthetase, *M. mobile* LeuRS (*Mm*LeuRS), which also naturally lacks editing domain, exhibits identical AMP formation activity at absence or presence of cognate tRNA^Leu^ (41). Furthermore, cognate tRNA is unable to stimulate any AMP formation for the editing domain-deprived *E. coli* LeuRS (*Ec*LeuRS-*Mm*Linker) (41). Therefore, the observed increase in AMP formation activity of *Sc*mtThrRS after tRNA^Thr^2 addition is significant and really reflects the tRNA-dependent pre-transfer editing of *Sc*mtThrRS, which is defective in post-transfer editing. To our knowledge, this is the first report describing such tRNA-isoacceptor specificity at the pre-transfer editing level. However, as reported for *Ec*ThrRS (30), *Sc*cyt*ThrRS* (19) and other systems (42), pre-transfer editing alone is not sufficient to prevent synthesis of mis-charged tRNAs. Therefore, as an error-prone synthetase, *Sc*mtThrRS readily accumulates Ser-tRNA^Thr^ under *in vitro* conditions. It is possible that synthesized Ser-tRNA^Thr^ is not detrimental to the mitochondrial translational machinery because of efficient discrimination by its elongation factor or the ribosome as is the case in *S. cerevisiae* cytoplasm (19). Alternatively, the yeast mitochondrion tolerates amino acid mis-incorporation to a certain extent. Indeed, under some conditions, tRNA mis-charging and amino acid mis-incorporation provides evolutionary benefits to bacteria, yeast or humans (43,44).

Our study also showed that tRNA^Thr^1 has the intrinsic capacity to stimulate tRNA-dependent pre-transfer editing activity according to the presence of the editing domain added in the chimeric enzyme CmThrRS. Indeed, it has been shown that the editing domain of bacterial ThrRS provides a binding interface for the minor groove of the tRNA acceptor stem and that deletion of the editing domain results in a dimeric enzyme that retains full activity in the activation step, while it is less efficient in the tRNA charging step (37). Similarly, we have previously found that deleting the N-terminal editing domain from yeast ThrRS results in an aminoacylation-impaired mutant; furthermore, amino acid alteration in the editing domain has an obviously negative effect on tRNA-dependent pre-transfer editing by yeast *Sc*cyt*ThrRS* (19). Collectively, these data indicate the critical contribution of the editing domain to aminoacylation and tRNA-dependent pre-transfer editing, possibly mediated by binding the acceptor stem of tRNA. The stimulation effect of chimeric CmThrRS observed here strongly suggests that interaction with the added editing domain may stabilize tRNA^Thr^1 in a conformation that is suitable for pre-transfer editing.

For tRNA^Thr^2-dependent pre-transfer editing, we found that both G35 and U36, but not A73 are key positive determinants. This is consistent with the cytosolic *Sc*cyt*ThrRS*, which depends critically on recognition of the anticodon bases for quality control (19). However, for the tRNA^Thr^1-dependent pre-transfer editing catalyzed by the chimeric CmThrRS, we showed the critical importance of different bases in the anticodon loop, such as U33a and G36, which suggests differences in the anticodon loop-binding mode between the two tRNAs during editing.

### tRNA-dependent pre-transfer editing is likely to occur at the aminoacylation active site

The location of the tRNA-dependent pre-transfer editing site has been debated. Preliminary evidence indicated that tRNA-dependent pre-transfer editing takes place in the editing domain, which is also the site of the post-transfer editing reaction. Indeed, fluorescence translocation-based assays combined with structure-directed mutagenesis in the CP1 editing domain of class Ia isoleucyl-tRNA synthetase (IleRS) showed that the tRNA^Ile^-dependent hydrolysis of Val-AMP occurs in the CP1 domain (10–11,16). This observation was consistent with the X-ray crystal structures showing that both the substrate of pre- and post-transfer editing bind the CP1 editing site with overlapping sites in LeuRS (12). In addition, a potential translocation channel was detected between the enzyme and tRNA, which could explain the migration of the adenylate molecules from the aminoacylation synthetic active site to the editing site (14,15). However, several reports indicated that tRNA-dependent pre-transfer editing occurs in the aminoacylation domain. This was first observed for GlnRS, a class I enzyme naturally devoid of a specialized editing domain, but able to catalyze tRNA-dependent aminoacyl-adenylate hydrolysis in the presence of a tRNA analog (17). Similarly, covalent inactivation of the *E. coli* LeuRS editing site by compound AN2690 did not reduce tRNA-dependent pre-transfer editing, indicating that the synthetic site was likely to be involved in tRNA-dependent pre-transfer editing (42). Furthermore, *Sc*cyt*ThrRS*-H151A/H155A, which harbored a defective editing domain, obviously catalyzed tRNA-dependent pre-transfer editing (19). However, these examples are subject to the criticism that analogs, inhibitors or mutations never mediate complete and definitive inactivation of the editing site, leading to careful and cautious interpretations. In the present study, we showed that *Sc*mtThrRS, a naturally occurring enzyme without a post-transfer editing domain, catalyzes significant tRNA^Thr^2-dependent pre-transfer editing; therefore, representing a perfect model to study the mechanism of tRNA-dependent pre-transfer editing without contaminating post-transfer editing activity. In addition, it directly suggests that, at least for *Sc*mtThrRS, tRNA-dependent pre-transfer editing takes place in the aminoacylation domain where the cognate and non-cognate adenylate molecules are synthesized.

### Specific binding mode of mitochondrial tRNA^Thr^1 to *Sc*mtThrRS

As stated previously, many yeast mitochondrial ThrRSs lack editing domains, suggesting that the loss of the editing domain occurred before the divergence of these species. Strikingly, both *S. pombe* and *C. albicans* mitochondria have retained canonical tRNA^Leu^(CUN) to decode CUN codons as Leu. *S. cerevisiae* mitochondria have lost tRNA^Leu^(CUN) and evolved tRNA^Thr^1 to decode CUN codons as Thr, yet, the advantage of this codon reassignment remains elusive. Despite high sequence similarity of *Sc*mtThrRS with *Sp*mtThrRS and *Ca*mtThrRS, the latter two ThrRSs failed to aminoacylate tRNA^Thr^1 both *in vitro* and *in vivo*. Other ThrRSs, such as *Ec*ThrRS and *Sc*cyt*ThrRS*, are also unable to charge tRNA^Thr^1 *in vitro* and *in vivo*. Furthermore, hcThrRS and hmtThrRS did not complement for the loss of *Sc*mtThrRS *in vivo*, which is likely to be due to an inability to charge tRNA^Thr^1. Therefore, *Sc*mtThrRS must have evolved tRNA^Thr^1-specific recognition elements. A crystal structure-based Ala-scanning mutagenesis strategy targeting all potential arginine or lysine residues has been employed to identify specific recognition sites for tRNA^Thr^1 in the anticodon binding domain of *Sc*mtThrRS (29). Among them, mutant R434A displayed specifically reduced affinity for tRNA^Thr^1, suggesting that this residue is a critical element of tRNA^Thr^ discrimination. However, Arg^434^ is a highly conserved residue present in nearly all ThrRSs (Supplementary Figure S1), including *Ec*ThrRS, *Sc*cyt*ThrRS*, *Sp*mtThrRS, *Ca*mtThrRS, hcThrRS and hmtThrRS, all of which were unable to charge tRNA^Thr^1. This suggests that Arg^434^ is only one element in a tRNA^Thr^ discrimination network, the whole process of which is probably more complex than expected. Our *in vivo* data generated with chimeric enzymes showed that the acquisition of tRNA^Thr^1 aminoacylation required the presence of both yeast mitochondrial aminoacylation and tRNA binding domains, suggesting that both domains co-evolved to follow the CUN codon reassignment and recognition of the new anticodon loop. Therefore, the strategy of Ala-scanning of amino acid targets may be extended to the aminoacylation domain. In this study, we observed another remarkable difference characterizing the two tRNA^Thr^ isoacceptors. While tRNA^Thr^2 spontaneously catalyzes tRNA-dependent pre-transfer editing, tRNA^Thr^1 required the artificial presence of an editing domain to stimulate pre-transfer editing. As the editing and aminoacylation domains usually clamp the acceptor stem of tRNA^Thr^, this strongly suggests differences in the interaction of the acceptor end of the two tRNAs with the aminoacylation domain. This also implies the existence of tRNA^Thr^ isoacceptor-specific interaction elements with the aminoacylation domain. Further peptide swapping and site-directed point mutagenesis will be performed in this domain to identify residues that potentially interact with tRNA^Thr^1 and may be part of the specific subset of amino acids involved in the recognition process.

## SUPPLEMENTARY DATA

Supplementary Data are available at NAR Online.

SUPPLEMENTARY DATA
