# Supplementary material for: A minimalist mitochondrial threonyl-tRNA synthetase exhibits tRNA-isoacceptor specificity during proofreading
Source: Nucleic Acids Res. 2014 Nov 20;42(22):13873–86. doi: 10.1093/nar/gku1218 (PMC4267643; doi:10.1093/nar/gku1218)
Supplement: SUPPLEMENTARY DATA [file supp_42_22_13873__index.html]

A minimalist mitochondrial threonyl-tRNA synthetase exhibits tRNA-isoacceptor specificity during proofreading — A minimalist mitochondrial threonyl-tRNA synthetase exhibits tRNA-isoacceptor specificity during proofreading — SUPPLEMENTARY DATA 

# A minimalist mitochondrial threonyl-tRNA synthetase exhibits tRNA-isoacceptor specificity during proofreading

## SUPPLEMENTARY DATA

**Files in this Data Supplement:**

- SUPPLEMENTARY DATA
