# Supplementary material for: A minimalist mitochondrial threonyl-tRNA synthetase exhibits tRNA-isoacceptor specificity during proofreading
Source: Nucleic Acids Res. 2014 Nov 20;42(22):13873–86. doi: 10.1093/nar/gku1218 (PMC4267643; doi:10.1093/nar/gku1218)
Supplement: SUPPLEMENTARY DATA [file supp_gku1218_nar-02394-v-2014-File002.docx]

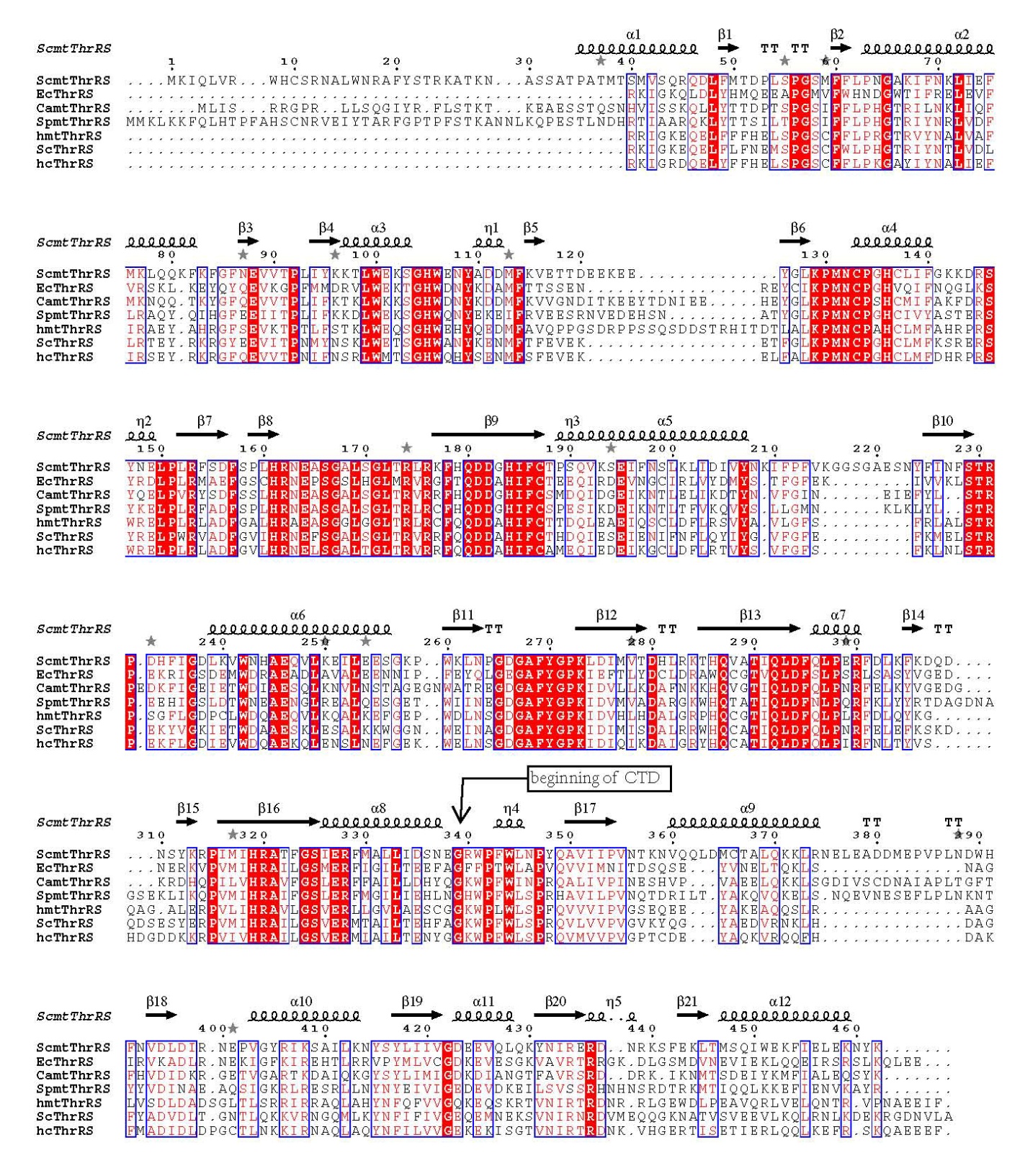


**Supplementary Figure 1. Primary sequence alignment of various ThrRSs.**

*Sc*mtThrRS, *Sp*mtThrRS, *Ca*mtThrRS precursors, aminoacylation and CTDs from various ThrRSs were aligned with secondary structure elements indicated on the top. The beginning of CTD of various ThrRSs was marked.

**
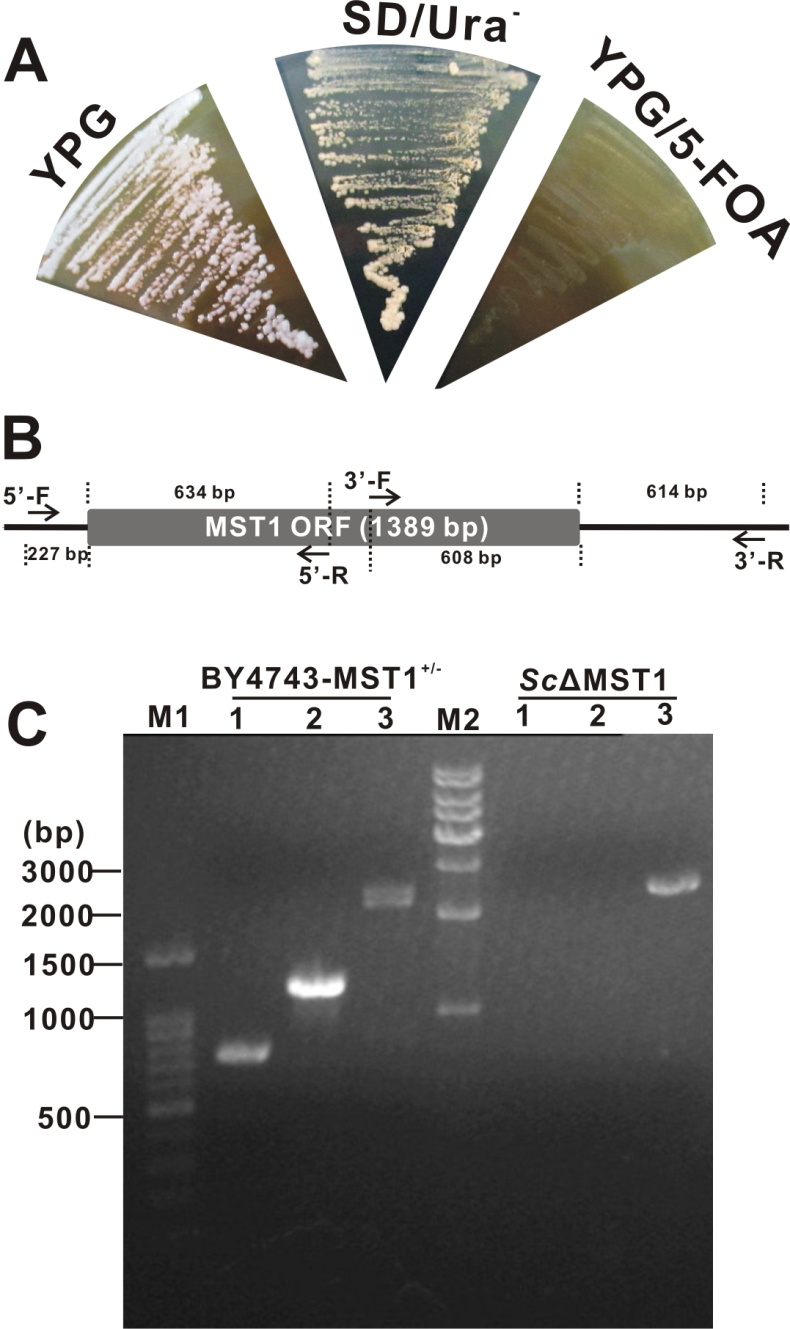
**

**Supplementary Figure 2. Construction and characterization of the *Sc*Δ*MST1* strain.**

(**A**) Growth phenotype of constructed knockout strain on YPG, SD/Ura^-^ and YPG/5-FOA plates. (**B**) Four primers designed based on the *MST1* ORF and its upstream or downstream sequences. 5’-F (5’gaggctgatcttgatcaag3’) and 3’-R (5’ctgaagaagaggttcaacac3’) primers were in the upstream and downstream, respectively; while 5’-R (5’gaacttccttagtctagtc3’) and 3’-F (5’aactgaaccctggtgatggt3’) were in the ORF. Relative location of four primers was indicated. (**C**) PCR-based confirmation of MST1 knockout. 100 bp ladder marker (M1) and 1000 bp ladder marker (M2) were loaded for size identification. Representative fragment sizes were indicated on the left based on two markers. Fragments from 5’-F/5’-R or 3’-F/3’-R or 5’-F/3’-R using genome of either diploid or *Sc*ΔMST1 were indicated as 1, 2 or 3, respectively. With genomic DNA from the diploid strain BY4743-MST1^+/-^ as template, one fragment was amplified with the expected size of 861 bp (lane 1: 227+634bp) using 5’-F/5’-R primers. Using 3’-F/3’-R primers a fragment of 1222 bp (lane 2: 608+614bp) was amplified. Using 5’-F/3’-R primers, two fragments of 2230 bp (from the chromosome containing wild-type *MST1* gene) and 2450 bp (from the chromosome containing *MST1* knockout by the kanamycin resistance gene) was obtained (lane 3). In contrast, when genomic DNA from the haploid strain *Sc*ΔMST1 was used as template, no fragments were obtained with 5’-F/5’-R primers or 3’-F/3’-R primers and only one fragment with the size of 2450 bp was amplified (lane 3), confirming that the haploid strain harbored *MST1* replaced by kanamycin resistance gene.


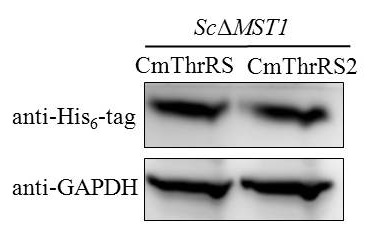


**Supplementary Figure 3. Protein level of CmThrRS andCmThrRS2 in the *Sc*Δ*MST1* strain.**

*Sc*Δ*MST1* transformant harboring p425TEF-MTS-CmThrRS or p425TEF-MTS-CmThrRS2 was cultured and harvested by centrifugation at 3000 ×g for 5 min at 4°C. Cells were resuspended in ice-cold lysis buffer containing 50 mM sodium phosphate (pH 8.0), 1 mM EDTA, 300 mM NaCl, 1 mM PMSF, 10% glycerol and lysed in a glass beads grinder at 4°C. Proteins were separated by 10% SDS-PAGE gel electrophoresis and the transferred membrane was immunoblotted with either anti-His_6_ antibody (M20001, Abmart, China) or anti-GAPDH antibody (M20028, Abmart, China).
